# Pruritus is common in patients with chronic liver disease and is improved by nalfurafine hydrochloride

**DOI:** 10.1038/s41598-021-82566-w

**Published:** 2021-02-04

**Authors:** Shuhei Yoshikawa, Takeharu Asano, Mina Morino, Keita Matsumoto, Hitomi Kashima, Yudai Koito, Takaya Miura, Yuko Takahashi, Rumiko Tsuboi, Takehiro Ishii, Haruka Otake, Junichi Fujiwara, Masanari Sekine, Takeshi Uehara, Kazuhito Yuhashi, Satohiro Matsumoto, Shinichi Asabe, Hiroyuki Miyatani, Hirosato Mashima

**Affiliations:** Department of Gastroenterology, Saitama Medical Center, Jichi Medical University, 1-847 Amanuma-cho, Omiya-ku, Saitama, 330-8503 Japan

**Keywords:** Gastroenterology, Hepatology

## Abstract

Pruritus is known to be a common complication in hepatitis patients, but the exact frequency and degree are not fully elucidated. Thus, we evaluated pruritus of 450 patients with chronic liver disease at our hospital. Pruritus was observed in 240 (53%) of the patients. Pruritus was significantly associated with males (OR = 1.51, *P* = 0.038) and patients with alkaline phosphatase (ALP) ≥ 200 U/L (OR = 1.56, *P* = 0.0495) and was significantly less in HBsAg-positive patients (OR = 0.449, *P* = 0.004). Seasonally, there was no difference in the frequency of pruritus between summer and winter. Of the 24 refractory pruritus patients treated with nalfurafine, 17 (71%) indicated improvement of itch, which is defined as a decrease in the visual analog scale score ≥ 30 mm. Pruritus was improved by nalfurafine both during daytime and nighttime in the Kawashima’s scores evaluation. All patients who received nalfurafine exhibited improved Kawashima’s scores ≥ 1 point during the daytime or nighttime. In conclusion, pruritus occurred in > 50% of patients with chronic liver disease, and predictors of pruritus were males and ALP ≥ 200 U/L. Nalfurafine may be useful for pruritus, regardless of whether daytime or nighttime.

## Introduction

Pruritus is a common comorbidity in chronic liver disease and kidney disease^1^. These patients frequently complain of pruritus despite having no rash or skin findings. Patients with chronic liver disease develop systemic itch that significantly impairs activity and sleep. Often, it is not relieved by scratching itchy areas of the skin. Patients with primary biliary cholangitis (PBC) frequently suffer from pruritus in chronic liver disease^2–5^. The prevalence of pruritus has been reported to be 70% in patients with PBC. In addition, pruritus has been reported in 8% of patients with chronic hepatitis B^6^ and in 2.5–20% of patients with chronic hepatitis C^7,8^. Cholestasis due to hepatitis, cirrhosis, or obstructive jaundice causes itching. Although bile acids have been suggested to be a factor in pruritus^9^, no correlation has been found between the intensity of pruritus and bile acid concentrations in the skin or serum^10,11^. Recently, lysophosphatidic acid and autotaxin, which controls its production, have been suggested to be involved in the development of itching^12,13^, but the underlying mechanism of the itch due to cholestasis is unknown. Generally, itching mechanisms are classified into peripheral pruritus and central pruritus caused by chronic renal failure (hemodialysis), bile stasis, and liver damage^14^. Peripheral pruritus is mainly due to histamine release from mast cells, which stimulates the itch receptor at the epidermal-dermal junction. In central pruritus, β-endorphin, Met-enkephalin, and endomorphin-1,2, μ-receptor agonists, which are endogenous opioids of the pruritus induction system, are released, and μ receptors in nerve tissue are stimulated^15,16^. Activation of μ receptors causes itching. In patients with cirrhosis, the concentration of plasma Met-enkephalin, an endogenous opioid activating µ receptor, was significantly increased relative to that in healthy subjects^17^.

Pruritus may be a major problem in patients’ quality of life, so its management is very important in the treatment of liver disease. However, few detailed studies have examined the relationship between liver disease status and pruritus. Among chronic liver disease patients with pruritus, antihistamines, sedatives, and topical drugs are effective in only a few cases, and the condition is often treatment-resistant pruritus. Regarding seasonal fluctuations, it has been reported that itching worsens in winter or summer, but the existence and degree of fluctuations are not fully understood. Therefore, there is an urgent need to identify patient characteristics, pruritus-associated factors, and timing of patients who at risk of pruritus.

Nalfurafine hydrochloride, which is a κ-opioid receptor agonist, suppresses pruritus by activating κ receptors that act on pruritus suppression with endogenous opioids. Recently, nalfurafine has been reported to have an excellent therapeutic effect on pruritus caused by liver disease resistant to existing treatments^4,18^. However, reports on the actual frequency and extent of effects are scarce.

The purpose of this study is to evaluate the actual condition of pruritus in chronic liver disease, such as frequency, degree, and response to treatment. We administered a questionnaire survey to chronic liver disease patients to confirm the current status of the complication rates and degree of pruritus. We also examined the relationship between blood sampling data/patient background factors and pruritus and then assessed whether any of these factors were associated with pruritus. In addition, we examined the efficacy of nalfurafine for the treatment of existing treatment-resistant pruritus in patients with chronic liver disease.

## Methods

### Study population

Four hundred and fifty outpatients with chronic liver disease who visited the Gastroenterology Department of Saitama Medical Center, Jichi Medical University, from November 2017 to January 2018, were included in this study. We performed a blood biochemical evaluation of the patient. Regarding etiology of their chronic liver disease, hepatitis B virus (HBV) patients were positive for hepatitis B surface antigen (HBsAg), hepatitis C virus (HCV) patients were HCV-RNA positive or after HCV eradication. Patients with PBC, autoimmune hepatitis (AIH), and non-alcoholic steatohepatitis (NASH) had been proven by liver biopsy. Alcoholic patients were defined as the patients consumed ethanol over 60 g/day without viral infection or autoantibodies. Liver fibrosis was assessed based on laboratory data using the FIB-4 index^19^. Hepatic reserve function was evaluated by modified albumin-bilirubin (ALBI) grade^20^.

We administered a questionnaire to these patients about the severity of their itch. Based on the score of Kawashima et al.^21^, the questionnaire contained questions about the presence or absence of pruritus, degree of pruritus, time of day when pruritus was experienced, use of a therapeutic agent for pruritus, and achievement of treatment-related symptom improvement. We analyzed the associations between questionnaire results and patients’ blood test and background factors. All patients provided informed consent to participate in this study. All clinical procedures were carried out in accordance with the Good Clinical Practice Guidelines and the Declaration of Helsinki. This study was approved by the institutional review board of Saitama Medical Center, Jichi Medical University.

### Evaluation of seasonal changes in itch

We first distributed a questionnaire on pruritus in the winter (November 2017 to January 2018) and received answers from 450 patients. Then, we distributed the same questionnaire to the 450 patients a second time in the summer (from July 2018 to September 2018), and responses were received from 303 patients. We investigated the differences in itch severity between summer and winter in 303 patients. Furthermore, we compared patients who had pruritus all year with other patients and examined the relationships between patients’ blood test/background factors and pruritus.

### Treatment efficacy of nalfurafine

For patients with pruritus, we first instructed skin care and prescribed topical medications. We then prescribed oral antihistamines and antiallergic drugs to those who still had itching. After that, we defined refractory pruritus patient as a patient who still had pruritus even after sufficient pre-existing treatment.

We prescribed nalfurafine hydrochloride for 24 patients with refractory pruritus. Patients with severe liver dysfunction (Child C) were excluded. Nalfurafine hydrochloride 2.5 μg (REMITCH Capsules, Toray Industries, Inc. and Sumitomo Dainippon Pharma Co., Ltd., Tokyo, Japan) was orally administered once daily before bedtime for 12 weeks. We evaluated the curative effect by using the Kawashima et al. proposed score^21^ and Visual Analog Scale (VAS) score^22^. Kawashima’s criteria of pruritus severity was rated on a five-point scale ranging from Grade 4 (Very severe, intolerable itch, or interfering with sleep due to itch) to Grade 0 (little or no itch). VAS score showed quantitatively the degree of pruritus from 0 mm (no itch) to 100 mm (maximum itch), according to the patients’ subjective perception of pruritus severity. We defined treatment efficacy as a VAS reduction of ≥ 30 mm. In a retrospective analysis, we investigated the relationship between pruritus and data from patients with refractory pruritus treated with nalfurafine.

### Statistics

Data was expressed as median (range) values. Univariate and multivariate logistic regression analyses were employed to determine the independent factors that could significantly predict pruritus. We also calculated the odds ratios (OR) and 95% confidence intervals. Comparison between the three groups was performed by the Fisher's PLSD method. Statistical significance was set at *P* < 0.05. All statistical analyses were performed with EZR version 1.50 (Saitama Medical Center, Jichi Medical University, Saitama, Japan)^23^, which is a graphical user interface for R (The R Foundation for Statistical Computing, Vienna, Austria, version 2.13.0).

## Results

### Patients’ characteristics

Four hundred and fifty patients with chronic liver disease were included in this study. The patients’ characteristics and laboratory data at the evaluation of pruritus are presented in Table 1. The subjects included 189 men and 261 women, and the median age was 68 years (18–87 years). The most common liver background of the patients was hepatitis C in 186 (41%) patients. Ninety patients had a history of hepatocellular carcinoma. Pruritus was observed in 240 (53%) patients. Among pruritus patients, the degree of pruritus was 191(80%) patients with mild, and 42 (18%) patients suffered from moderate to severe pruritus, which interfering with daily activities. Regarding the time of day when pruritus was experienced, 32 (13%) patients had pruritus in the daytime, 133 (55%) patients in the nighttime, and 71 (30%) patients had pruritus in both daytime and nighttime.

**Table 1:** Patient background and laboratory data at the evaluation of pruritus in 450 patients with chronic liver disease.

Sex (male/female)	189/261
Age (years)	68 (18–87)
Etiology (HBV/HCV/Alc/NASH/PBC/AIH/other)	67/186/38/47/44/75/17
History of HCC (present/absent)	90/360
Alb (g/dL)	4.2 (2.3–5.2)
T-Bil (mg/dL)	0.65 (0.19–3.5)
AST (U/L)	27 (8–186)
ALT (U/L)	21 (6–261)
LDH (U/L)	210 (132–466)
ALP (U/L)	257 (87–1808)
γ-GTP (U/L)	30.5 (8–856)
Platelet count (× 10^4^/µL)	16.9 (2.3–45.4)
FIB-4 index	2.55 (0.29–22.93)
ALBI grade 1/2a/2b/3	315/68/61/6

Laboratory data are expressed as median (range) values.

*AIH* autoimmune hepatitis, *HBV* hepatitis B virus, *HCV* hepatitis C virus, *Alc* alcoholic hepatitis, *NASH* non-alcoholic steatohepatitis, *PBC* primary biliary cholangitis, *HCC* hepatocellular carcinoma, *ALBI grade* albumin–bilirubin grade.

### Factors associated with pruritus

**Figure 1:**
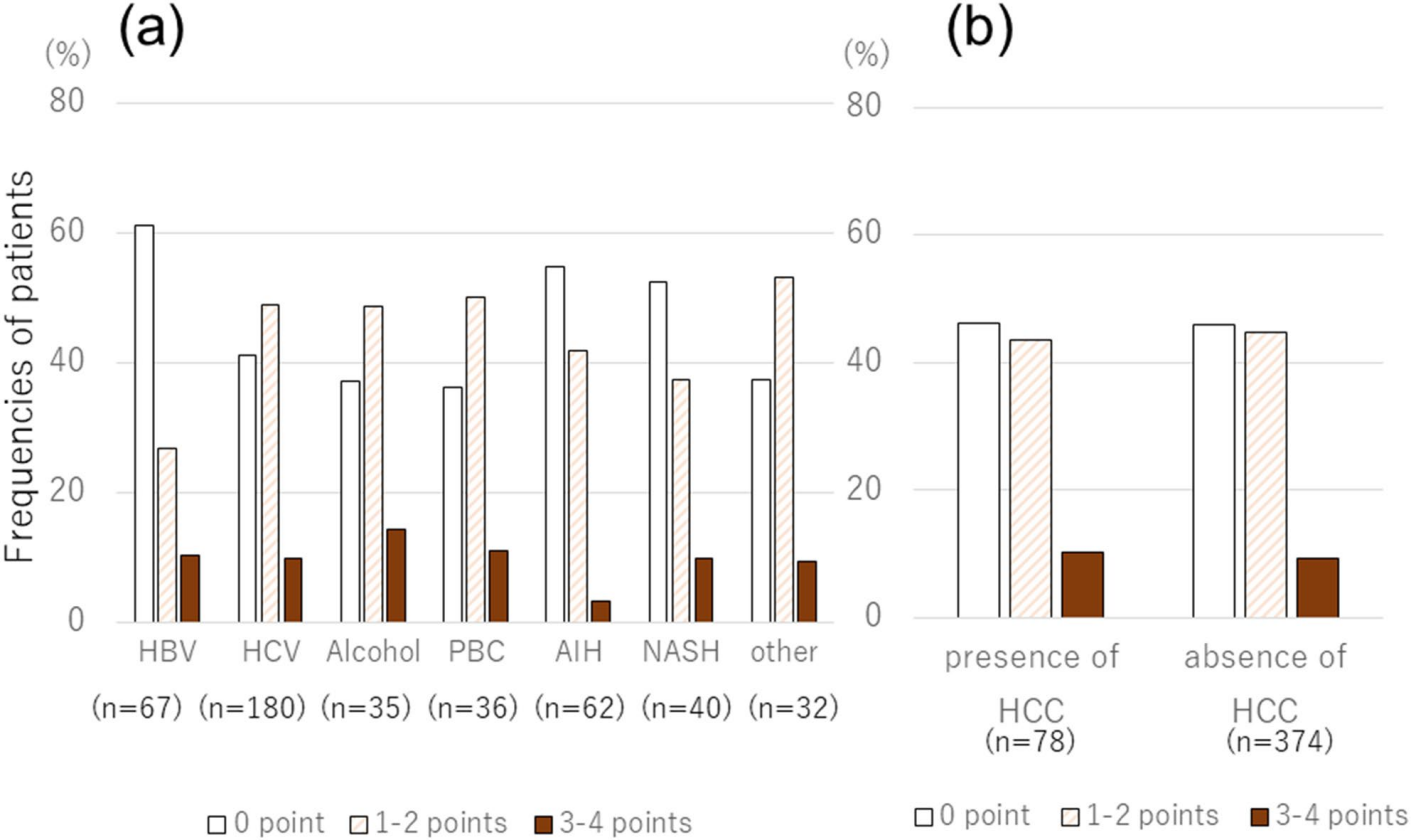
(**a**) The distribution of pruritus scores is shown, according to the etiology of chronic liver disease. HBsAg-negative status (*P* = 0.004) as factors significantly associated with pruritus in multivariate analysis. Severity of pruritus scores was not significantly different between diseases. (**b**) The distribution of pruritus scores is shown, according to the presence of hepatocellular carcinoma (HCC). There was no significant difference in the incidence or degree of pruritus regarding the presence or absence of HCC.

We evaluated the incidence and degree of pruritus for each disease (Fig. 1a). HBsAg-negative status (*P* = 0.004) as factors significantly associated with pruritus in multivariate analysis. Severity of pruritus scores was not significantly different between diseases. There was no significant difference in the incidence or degree of pruritus regarding the presence or absence of hepatocellular carcinoma (HCC) (Fig. 1b).

In univariate analysis, sex (male), HBsAg-negative status, high alkaline phosphatase (ALP) level (≥ 200 U/L), and low platelet count (≤ 10^4^/µL) were found to be significant factors associated with pruritus. The above four variables were used in multivariate analysis, which identified sex (male, OR = 1.51, *P* = 0.038), HBsAg-negative status (negative for hepatitis B, OR = 0.449, *P* = 0.004), and high ALP level (≥ 200 U/L, OR = 1.56, *P* = 0.0495) as factors significantly associated with pruritus (Table 2).

**Table 2:** Factors associated with pruritus in 450 patients with chronic liver disease.

	Odds ratio	(95% CI)	*P* value
HBsAg-positive	0.449	(0.259–0.778)	0.0043
ALP ≥ 200 (U/L)	1.560	(1.000–2.420)	0.0495
Sex (male)	1.510	(1.020–2.240)	0.0381

### Seasonal variation in pruritus

Of the 303 patients who had answered the same pruritus questionnaire during both summer and winter, 139 (46%) patients in summer and 159 (52%) patients in winter had pruritus. The blood test and background factors of the patients in whom pruritus was observed both in summer and winter were analyzed. Among these factors, univariate analysis only identified sex (male, *P* = 0.0094) as a significant factor associated with pruritus. No significant factor was found in the multivariate analysis.

We then compared potential factors associated with pruritus among three groups: group 1 (n = 106), no itching in summer or winter; group 2 (n = 96), itching in either summer or winter; and group 3 (n = 101), itching in both summer and winter. Although there was a significant difference in sex (male) between groups 1 and 3 (*P* = 0.026) and between groups 2 and 3 (*P* = 0.0218), there were no significant differences in the other background factors among the three groups.

### Efficacy of nalfurafine for pruritus

**Figure 2:**
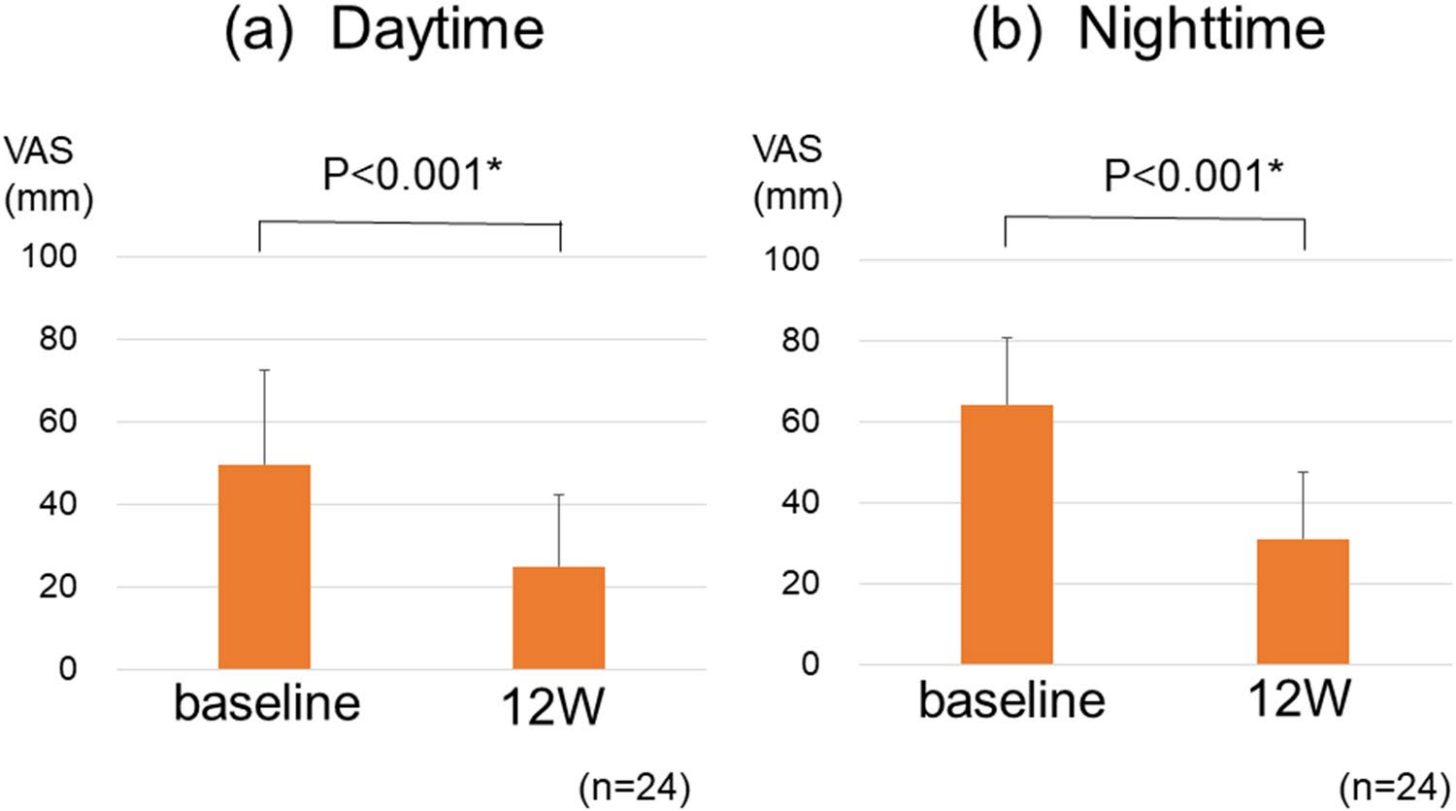
(**a**) Visual Analog Scale (VAS) scores significantly decreased 12 weeks after administration of nalfurafine when compared with baseline scores during daytime. The average VAS score improved from 50 to 25 mm (*P* < 0.001). (**b**) VAS scores significantly decreased 12 weeks after administration of nalfurafine when compared with baseline scores during nighttime. The average VAS score improved from 64 to 31 mm (*P* < 0.001). The error bars represent standard errors.

Twenty-four patients with refractory pruritus were treated with nalfurafine, and the background and data were presented in Table 3. Of these patients, all used topical antipruritic medications and 14 (58%) took antihistamines. Regarding the efficacy of nalfurafine in patients with treatment-resistant pruritus, the average VAS score improved significantly from 50 to 25 mm in daytime (*P* < 0.001) and from 64 to 31 mm in nighttime (*P* < 0.001) (Fig. 2). Of the 24 patients who received nalfurafine, 17 (71%) indicated improvement of itch, which is defined as a decrease in VAS score ≥ 30 mm. The VAS value and Kawashima’s score were well correlated (*P* < 0.001). The VAS values of kawashima’s scores 0, 1, 2, 3 and 4 were 5.8 ± 7.4 mm, 18.6 ± 15.7 mm, 38.9 ± 19.3 mm, 59.5 ± 26.7 mm and 74.6 ± 21.6 mm, respectively.

**Table 3:** Patient background and laboratory data at the evaluation of pruritus in 24 patients with intractable pruritus.

Sex (male/female)	12/12
Age (years)	65.5 (33–81)
Etiology (HBV/HCV/Alc/NASH/PBC/AIH/other)	0/12/5/1/5/0/1
History of HCC (present/absent)	4/20
Alb (g/dL)	3.9 (2.1–4.9)
T-Bil (mg/dL)	0.7 (0.2–35.6)
AST (U/L)	28.5 (11–103)
ALT (U/L)	23 (5–114)
LDH (U/L)	224 (167–387)
ALP (U/L)	387 (166–1185)
γ-GTP (U/L)	50 (16–362)
Platelet count (× 10^4^/µL)	15.4 (4.2–40.4)
FIB4 index	2.40 (0.29–11.66)
ALBI grade 1/2a/2b/3	12/4/6/2

Laboratory data are expressed as median (range) values.

*AIH* autoimmune hepatitis, *HBV* hepatitis B virus, *HCV* hepatitis C virus, *Alc* alcoholic hepatitis, *NASH* non-alcoholic steatohepatitis, *PBC* primary biliary cholangitis, *HCC* hepatocellular carcinoma, *ALBI grade* albumin–bilirubin grade.

**Figure 3:**
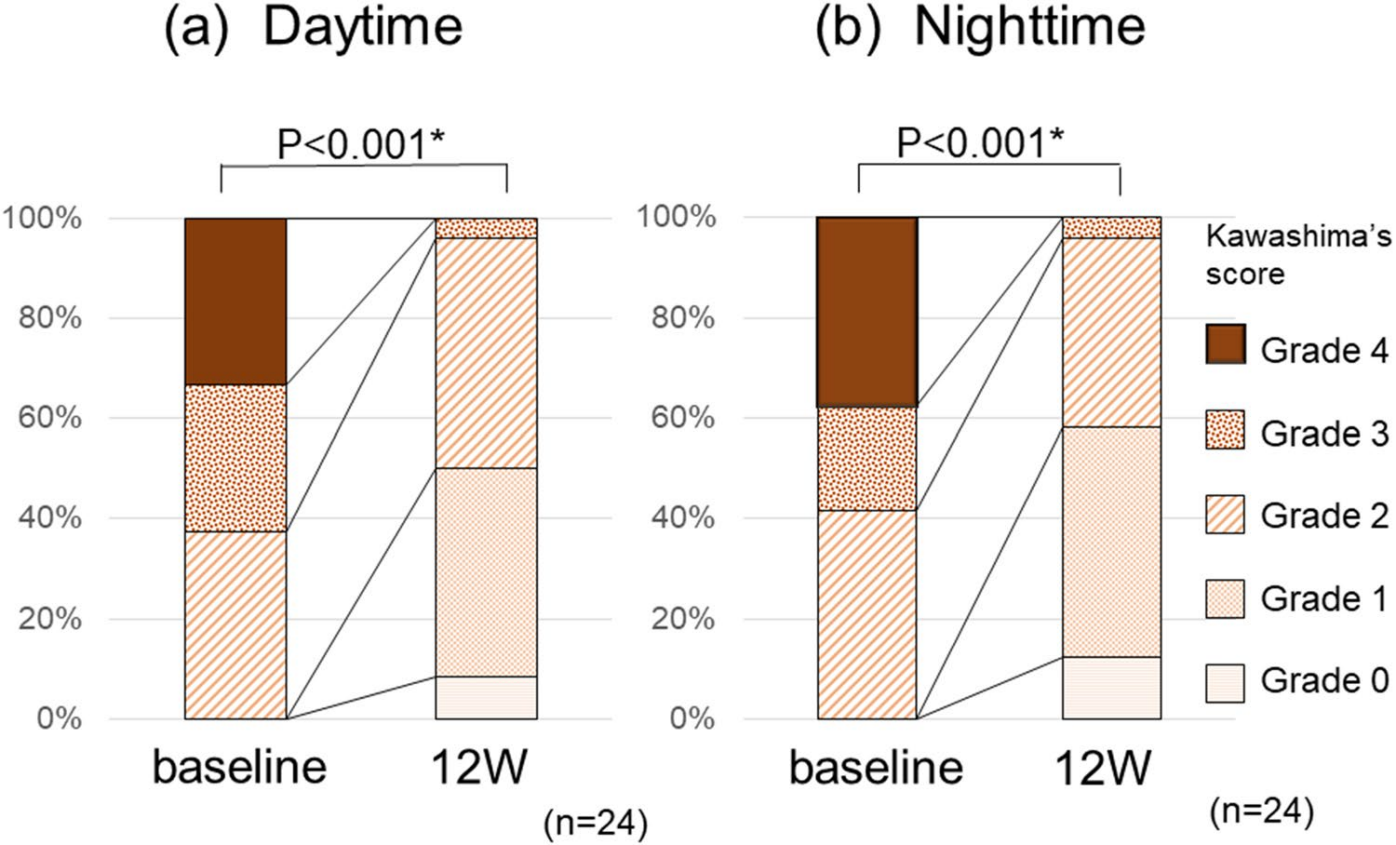
Kawashima’s score significantly decreased 12 weeks after administration of nalfurafine when compared with baseline scores during (**a**) daytime (*P* < 0.001) and (**b**) nighttime (*P* < 0.001).

The results for Kawashima’s score exhibited a significant improvement in pruritus after administration both during daytime and nighttime (Fig. 3). All patients who received nalfurafine exhibited improvement ≥ 1 point during the daytime or nighttime. No significant factors were found in the analysis of patient background and blood test data predicting patients with therapeutic effects. As a side effect, one patient (4%) felt mild sleepiness. No other obvious side effects were observed, including liver dysfunction.

## Discussion

In our study, pruritus was observed in more than half of the patients with chronic liver disease, was significantly associated with males and ALP ≥ 200 U/L , and significantly less common in patients positive for HBsAg (hepatitis B-positive patients). There was no difference in the frequency of pruritus between summer and winter in the seasonal variation analysis, and the numbers of patients with pruritus in both summer and winter were significantly higher in men. In addition, the efficacy of nalfurafine for patients with pruritus resistant to existing treatment was as high as 71%, and a significant improvement in the degree of pruritus was obtained both during daytime and nighttime.

In our institution, pruritus in patients with chronic liver disease was more common in men, but other reports indicate that women have more pruritus^24,25^. It is known that PBC is more common in women and that pruritus caused by skin disease is more common in women^26,27^. In the present study, there were relatively few patients who had cirrhosis associated with PBC or AIH, and more patients with cirrhosis due to hepatitis C virus and alcohol in men. The reason why pruritus was significantly lower in hepatitis B-positive patients may be that these patients were relatively younger than the other patients^28^.

It is known that cholestasis causes pruritus, and our study also revealed a significant difference in ALP values, suggesting the effect of cholestasis. The detailed mechanism by which pruritus increases in patients with high ALP levels has not been elucidated, so future studies may be necessary. Significant differences in pruritus were reported in patients with hepatitis C, depending on the value of γ-glutamyl transpeptidase (γ-GTP)^29^. But in our study, no significant difference in γ-GTP values was observed, probably because of the effects of drinking alcohol and fatty changes in the liver.

Several correlations between fibrosis markers and pruritus have been reported^28,29^. However, in our study, there was no significant correlation between the FIB4 index, an index of fibrosis, and pruritus. Thrombocytopenia was identified as a significant factor in our univariate analysis, but it was not significant in the multivariate analysis. Regarding the relationship between liver function and pruritus, no significant difference in Child–Pugh analysis results was reported in the previous reports, and the analysis by our study was conducted using the albumin–bilirubin (ALBI) score, but again, no significant difference in the results was found.

Regarding the seasonal variation, pruritus associated with general skin diseases has been reported to worsen in summer or winter^30,31^. Although it may be exacerbated by skin irritation associated with sweating in summer and dryness in winter, this study did not reveal any significant change in the frequency of pruritus associated with liver disease between summer and winter. Recurrence of pruritus has been reported after discontinuation of nalfurafine^32^, and some patients will experience pruritus regardless of the season. In both summer and winter, itching was significantly more common in men, but there was no significant difference in background liver or serum biochemistry factors other than sex (male), and the cause was not clear.

Japanese PBC clinical practice guideline 2017 recommend nalfurafine for PBC-related pruritus, along with cholestyramine, antihistamines^33^. Furthermore, the Japanese dermatological association's pruritus clinical practice guideline 2020 recommends the use of nalfurafine for intractable pruritus in patients with hemodialysis and patients with chronic liver disease^34^. Therefore, we defined refractory pruritus as a case where existing treatments such as antihistamine or antiallergic medicines are insufficient, similar to the pruritus clinical practice guideline.

In this study, the nalfurafine response rate to refractory pruritus was 71%, as assessed by VAS scores, and Kawashima’s score improved in all cases, indicating a very good response rate. Since itching at night also causes insomnia, itch control is directly linked to activities of daily living in patients with chronic hepatitis. According to this questionnaire, many patients were the first to recognize that the cause of itch was the liver, and it is thought that there is a high demand for the management of potential pruritus. Both liver cirrhosis and chronic hepatitis have symptoms that hinder activities of daily life, so it is important to obtain detailed information about symptoms and provide treatment to enhance patient satisfaction and quality of life.

Our study had certain limitations. First, this was a single-center study with a small number of patients, 10% of which had PBC (n = 44). Patients who were eligible for treatment with nalfurafine were resistant to existing treatments, and the number of treated patients was small. Second, the evaluation period was relatively short, about 12 weeks, and the long-term effects of nalfurafine were unclear. A large-scale, long-term, randomized, controlled trial should be performed to evaluate the efficacy of nalfurafine. Third, we did not make a direct comparison between nalfurafine and cholestyramine, which is the first choice for pruritus due to chronic liver disease in some guidelines, so we could not evaluate which one was more useful. The reactivity of cholestyramine to intractable pruritus has not been fully elucidated.

In conclusion, pruritus was observed in more than half of the patients with chronic liver disease at our hospital in this study. Pruritus was especially associated with males, hepatitis B virus-negative status, and high ALP level. The patients with chronic liver disease and existing treatment-resistant pruritus who were treated with nalfurafine had a high response rate and exhibited significant improvement in pruritus both during daytime and nighttime. Nalfurafine may improve the quality of life of patients with chronic liver disease by reducing the incidence of pruritus.

## Data Availability

We can provide adequate assurances that we can comply with the publication's requirements for sharing materials.
